# Quality of guidelines for the management and prevention of occupational risks during pregnancy: a systematic quality assessment using the Appraisal of Guidelines for Research and Evaluation (AGREE II)

**DOI:** 10.5271/sjweh.4323

**Published:** 2026-09-01

**Authors:** Martina Lupia, Ivan Solà, Rocío Villar, Marco Culqui, Pilar Díaz, Marilina Santero, Joan Mirabent, Fernando G Benavides, Consol Serra

**Affiliations:** 1 CiSAL-Center for Research in Occupational Health, Hospital del Mar Research Institute/Medicine and Life Sciences Department, Universitat Pompeu Fabra, Barcelona, Spain.; 2 Occupational Health Service, Hospital del Mar, Barcelona, Spain.; 3 Iberoamerican Cochrane Network, Barcelona, Spain.; 4 Institut de Recerca Sant Pau, Barcelona, Spain.; 5 CIBER de Epidemiología y Salud Pública (CIBERESP). Instituto de Salud Carlos III, Madrid, Spain.; 6 Pontificia Universidad Católica del Ecuador, Facultad de Salud y Bienestar, Quito, Ecuador.; 7 Facultad de Medicina, Unidad docente Hospital Universitario Sant Pau, Universidad Autónoma de Barcelona, Barcelona, Spain.

**Keywords:** control, occupational hazard, occupational health, pregnant worker, prevention, review, safety management, systematic review, work, work–life balance

## Abstract

**Objective:**

This review aimed to appraise the quality of existing guidelines on the management of occupational risks for employed women during pregnancy, identifying and describing their characteristics.

**Methods:**

We systematically identified guidelines in MEDLINE, Embase, CINAHL, UpToDate, GIN International Guidelines Library, ECRI Guidelines Trust and websites of institutions and scientific societies. Four reviewers independently appraised each included document using the Appraisal of Guidelines for Research and Evaluation (AGREE II), generating domain scores of 0–100% (lowest to highest quality). An overall quality score for each guideline was assigned to each document by consensus among reviewers.

**Results:**

The study included 31 documents: only 4 (12.9%) had good methodological quality for use in clinical practice; 27 (87.1%) scored poorly in domain III (rigor of development), failing to describe literature searches and formulation of recommendations methods, relationship between scientific evidence and recommendations or updating procedures. Domain IV (clarity of presentation) scores were higher as most documents presented their recommendations clearly, and 14 (45.1%) achieved scores of >60% for ease of identification and interpretation. Guidelines were generally clearer in defining their objectives, but none provided implementation tools.

**Conclusions:**

Guidelines for the management and prevention of occupational risks during pregnancy lacked sufficient rigor in their development. Despite generally clear objectives and presentation, critical gaps in methodology undermine their utility and applicability. There is a clear need to improve the development process of guidelines for the management and prevention of occupational risks during pregnancy.

Pregnancy represents a critical life stage that confers higher sensitivity and vulnerability from various factors, including occupational hazards. Pregnant workers may be exposed to workplace risks that adversely affect both maternal and fetal health. Recent systematic reviews demonstrate that psychosocial work stress significantly increases by 32% the risk of preterm birth (1), pre-eclampsia, and reduced birth weight (2). Biomechanical constraints such as prolonged standing, heavy lifting, and shift work are associated with adverse pregnancy outcomes (3, 4), while occupational exposure to chemical and biological agents poses documented risks of fetal loss and congenital abnormalities (5). In Spain, as in many other countries, legislation mandates the protection of pregnant women through risk prevention. The EU Pregnant Workers Directive (92/85/EEC) establishes a framework requiring employers to assess workplace risks and implement protective measures (6). The identification, characterization, and control of occupational risks constitute the foundation for prevention and protection of both mother and fetus in the workplace (7).

For helping decision-making at different levels including companies, scientific societies, governmental agencies, unions, and other organizations, it is essential to have guidelines based on rigorous methodology. Clinical practice guidelines are defined as “systematically developed statements to assist practitioner and patient decisions about appropriate health care for specific clinical circumstances” (8). The potential benefits of guidelines depend critically on their methodological quality (9). Therefore, appropriate methods and strategies in guideline development are essential for their implementation and effectiveness (10). Guideline development involves identifying and defining a topic area, forming a multidisciplinary expert panel, conducting a systematic review of the evidence, formulating evidence-based recommendations, and grading the strength of recommendations (11). The entire process must be transparent, evidence-based, and involve stakeholder’s participation (12).

In the case of pregnant workers, several entities address these issues by developing guidelines, including companies, scientific societies, governmental agencies, labor unions, and other organizations. Each of these stakeholders contributes with different perspectives to the shared objective of improving working conditions, balancing work and private life, and ensuring risk prevention and protection of pregnant women throughout gestation. Nevertheless, when knowledge or guidelines fail to provide concrete and clear answers, the “precautionary principle” is ultimately applied, which in this context translates to removal of the pregnant worker from her workplace (13, 14). In occupational medicine practice, when workplace hazards cannot be fully mitigated (primary preventive measure), physicians may recommend maternity leave or pregnancy benefits, which is separation from employment as a preventive measure. This removal can have significant implications for maternal and fetal health, as well as for the worker’s economic situation and professional career, the employing company and colleagues, and the entire society (15).

Adane et al (16) analyzed the extent to which evidence on occupational risks and preterm birth was reflected in current policies and showed that such policies only partially reflected the existing evidence on the relationship between both variables. Also, many documents were developed without rigorous methodologies and without involving multiple stakeholders. For this reason, they argued the need to develop evidence-based policies using rigorous methods.

In this context, and to the best of our knowledge, a systematic evaluation of the methodology of existing guidelines on the management of occupational risks during pregnancy has not been conducted. Given the documented poor adherence to existing legislation and guidelines (17), such an evaluation is critically needed. Our hypothesis is that the quality of existing guidelines on occupational risks and pregnancy is limited. The main objective of our study was to systematically appraise the quality of existing guidelines on this topic, identifying and describing their characteristics.

## Method

This study systematically identified practice guidelines regarding occupational risk management of employed women during their pregnancies and evaluated their methodological quality.

The types of documents considered for inclusion were practice guidelines, position statements or technical reports including recommendations, targeting women who were employed and became pregnant.

### Search methods for identification of documents

We designed a search strategy for MEDLINE, Embase and CINAHL up to November 2023, complemented by a focused update in February 2026. We combined database-specific controlled vocabulary and natural language to define a set of search terms in three blocks related to the relevant components of the review objective (ie, pregnancy, employment and recommendations) (supplementary material, www.sjweh.fi/article/4323, S1).

We also searched the GIN International Guidelines Library and the ECRI Guidelines Trust. Additionally, we screened the summary and recommendations of four relevant UpToDate topics: ‘Working during pregnancy’, ‘Occupational and environmental risks to reproduction in females: specific exposures and impact’, ‘Overview of occupational and environmental health’, and ‘Overview of occupational and environmental risks to reproduction in females’. We completed the search by browsing websites from institutions and scientific societies relevant in the field of gynecology, preventive medicine and public health, and occupational health (table 1).

**Table 1:** List of sources searched of websites from Institutions and Scientific Societies to identify eligible documents.

Region	Country	Institution/site
GLOBAL	(Global)	International Commission on Occupational Health (ICOH) International Labour Organization (ILO) World Health Organization (WHO)
NORTH AMERICA	Canada	Society of Obstetricians and Gynecologists of Canada (SOGC) Canadian Centre for Occupational Health and Safety (CCOHS)
	US	American College of Obstetricians and Gynecologists (ACOG) American College of Occupational and Environmental Medicine (ACOEM) US Centers for Disease Control and Prevention (CDC) US National Institute for Occupational Safety and Health (NIOSH) US Occupational Safety and Health Administration (OSHA) US Preventive Services Task Force (USPSTF)
	México	Consejo Nacional Mexicano de Medicina del Trabajo (CNMMT) Instituto Mexicano Seguro Social (IMSS) Secretaría de Salud del Gobierno de México
CENTRAL/ SOUTH AMERICA	Latin América	Asociación Latinoamericana de Salud Ocupacional (ASLSO)
	Costa Rica	Asociación Costarricense de Medicina del Trabajo (ACOMET)
	Perú	Ministerio de Salud del Gobierno Peruano
	Colombia	Ministerio de Salud y Protección Social de Colombia
	Guatemala	Asociación de Medicina del Trabajo de Guatemala (ASOMET)
	Venezuela	Sociedad Venezolana de Salud Ocupacional (SOVESO)
	Ecuador	Ministerio de Salud Pública de Ecuador
	Brazil	Ministério da Saúde do Brasil Associação Nacional de Medicina do Trabalho (ANAMT)
	Chile	Instituto de Salud Pública de Chile Sociedad Chilena de Medicina del Trabajo (SOCHMET)
	Argentina	Ministerio de Salud de Argentina Sociedad de Medicina del Trabajo de la Provincia de Buenos Aires (SMTBA)
EUROPE	Finland	Finnish Institute of Occupational Health (FIOH)
	Norway	Norwegian Association of Occupational Physicians (NAMF) Norwegian Labour Inspection Authority (Arbeidstilsynet) Norwegian National Institute of Occupational Health (STAMI)
	Denmark	Danish Society of Occupational and Environmental Medicine (DASAM) National Research Centre for the Working Environment, Denmark (NRCWE)
	Germany	Institute for Prevention and Occupational Medicine of the German Social Accident Insurance Bundesministerium für Familie, Senioren, Frauen und Jugend Federal Institute for Occupational Safety and Health (BAuA)
	The Netherlands	Nederlandse Vereniging voor Arbeids- en Bedrijfsgeneeskunde (NVAB) National Institute for Public Health and the Environment (RIVM)
	France	Agence Nationale Sécurité Sanitaire Alimentaire Nationale (ANSES) Haute Autorité de Santé (HAS) Société Française de Santé au Travail (SFST)
	Switzerland	Swiss Society for Occupational Health in Health Care Facilities (SOHF) Société Suisse de Médicine du Travail (SGARM/SSMT)
	UK	Health and Safety Executive (HSE) Royal College of Obstetricians and Gynaecologists (RCOG) Royal College of Physicians (RCP) National Institute for Health and Care Excellence (NICE) Society of Occupational Medicine (SOM)
	Ireland	Health and Safety Authority, Ireland Irish Society of Occupational Medicine (ISOM)
	Italy	Istituto Nazionale per l’assicurazione contro gli infortuni sul lavoro (INAIL)
	Greece	Hellenic Society of Occupational and Environmental Medicine (HSOEM)
	Portugal	Instituto da Segurança Social (ISS) Sociedade Portuguesa de Medicina do Trabalho (SPMT)
	Spain	Instituto Nacional de Seguridad y Salud en el Trabajo (INSST) Instituto Nacional de la Seguridad Social (INSS) Instituto Sindical de Trabajo, Ambiente y Salud (ISTAS) Asociación Española de Especialistas en Medicina del Trabajo (AEEMT) Asociación Nacional de Medicina del Trabajo en el Ámbito Sanitario (ANMTAS) Asociación de Mutuas de Accidentes de Trabajo (AMAT) Agència de Salut Pública de Barcelona (ASPB) Institut Català de la Salut (CatSalut) GuiaSalud Laneko Segurtasun eta Osasunerako Euskal Erakundea (OSALAN) Osakidetza INVASSAT-OPAC repository (Institut Valencià de Seguretat i Salut en el Treball)
OCEANIA	Australia	Australian Government Department of Health
	Australia and New Zeland	Australian and New Zealand Society of Occupational Medicine (ANZSOM) Royal Australian and New Zealand College of Obstetricians and Gynaecologists (RANZCOG)

The inclusion criteria were any document on occupational risks and their prevention in pregnant workers, regardless of publication year or continent, published in English, Spanish, Italian or Portuguese. The exclusion criteria were documents focused only on the puerperium and breastfeeding, antenatal or postnatal care, documents with general recommendations on occupational health or exposure or without recommendations, obsolete documents or those that overlapped with valid documents.

### Screening and data extraction

One researcher screened the search results and recorded all the eligible documents in a Microsoft Office Excel database. For each document, data on the title, leading organization, geographic region, scope and preliminary eligibility assessment was registered. For excluded documents, a reason was provided. Four other researchers with a background in occupational health discussed and agreed on inclusion eligibility.

### Quality assessment

The quality of the included documents was appraised using the Appraisal of Guidelines for Research and Evaluation (AGREE II) instrument (18) (agreetrust.org), which assesses the methodological rigor and transparency of guidelines development. AGREE II comprises 23 items that assess relevant aspects related to six domains (table 2). This instrument was originally developed for the evaluation of clinical practice guidelines; however, it includes generic items applicable to other fields. Indeed, the AGREE II instrument has already been used in the field of occupational health (19–22).

**Table 2:** List of AGREE II domains. Adapted from Brouwers et al (18).

Domain & description	Item	Specific question
**I. Scope and purpose:** Aim and scope of the guideline, the specific health questions, and the target population	1	The overall objective(s) of the guideline is (are) specifically described.
	2	The health question(s) covered by the guideline is (are) specifically described.
	3	The population (patients, public, etc.) to whom the guideline is meant to apply is specifically described.
**II. Stakeholder involvement:** Asking for the involvement of appropriate stakeholders and the extent to which it represents the views of its intended users	4	The guideline development group includes individuals from all the relevant professional groups.
	5	The views and preferences of the target population (patients, public, etc.) have been sought.
	6	The target users of the guideline are clearly defined.
**III. Rigor of development:** Appraising the methods used to conduct guidelines, including the synthesis of the evidence, the methods to formulate the recommendations, and to update them	7	Systematic methods were used to search for evidence.
	8	The criteria for selecting the evidence are clearly described.
	9	The strengths and limitations of the body of evidence are clearly described.
	10	The methods for formulating the recommendations are clearly described.
	11	The health benefits, side effects, and risks have been considered in formulating the recommendations.
	12	There is an explicit link between the recommendations and the supporting evidence.
	13	The guideline has been externally reviewed by experts prior to its publication.
	14	A procedure for updating the guideline is provided.
**IV. Clarity of presentation:** Structure, format and presentation of the guideline	15	The recommendations are specific and unambiguous.
	16	The different options for management of the condition or health issue are clearly presented.
	17	Key recommendations are easily identifiable.
**V. Applicability:** The extent to which the guidelines offer tools to implement their recommendations	18	The guideline describes facilitators and barriers to its application.
	19	The guideline provides advice or tools on how the recommendations can be put into practice.
	20	The potential resource implications of applying the recommendations have been considered.
	21	The guideline presents monitoring or auditing criteria.
**VI. Editorial independence:** The possible impacts of competing interests	22	The views of the funding body have not influenced the content of the guideline.
	23	Competing interests of guideline development group members have been recorded and addressed.

The AGREE instrument uses a 7-point Likert scale to rate the degree of agreement with each item (1=strongly disagree to 7=strongly agree). The ratings are then converted into a domain score, the range of which depends on the number of items included in each domain. Based on these ratings, each domain is scored obtaining a scaled score from 0% (lowest quality) to 100% (highest quality). In addition, AGREE II proposes an overall assessment score for each guideline, using a 7-point Likert scale, as a proxy measure of documents trustworthiness.

Four reviewers independently appraised each included document to increase reliability, according to AGREE II’s user manual (the evaluation must be carried out by at least two evaluators). Three of the reviewers had extensive backgrounds in the field of guidelines evaluation and one had expert knowledge in occupational health.

### Data analysis and synthesis

According to AGREE II, we calculated each domain score by summing up the ratings of all appraisers for each item within the corresponding domain and scaling the total as percentage of the maximum possible score for that domain. We calculated the scaled domain according to the formula (total domain obtained score – minps) / (maxps – minps) where maximum possible score (maxps)=7 (strongly agree) × domain items × number of appraisers and minimum possible score (minps)=1 (strongly disagree) × domain items × number of appraisers (supplementary material S2 and S3).

In addition, we conducted an overall assessment for each guideline, using a 7-point Likert scale (1=strongly disagree to 7=strongly agree). The overall quality score was obtained through consensus among the reviewers.

We summarized the characteristics from the included documents and recorded their scores for each AGREE II domain, as well as their overall assessment scores (18). We classified the guidelines from highest to lowest quality according to the scores obtained in the domains most closely related to their internal validity and usability (ie, rigor of development and clarity of presentation). To facilitate visual interpretation, we applied an ad hoc color-coding scheme to the domain scores to simulate a heat map, ranging from lower to higher quality.

## Results

### Search results and selection

**Figure 1:**
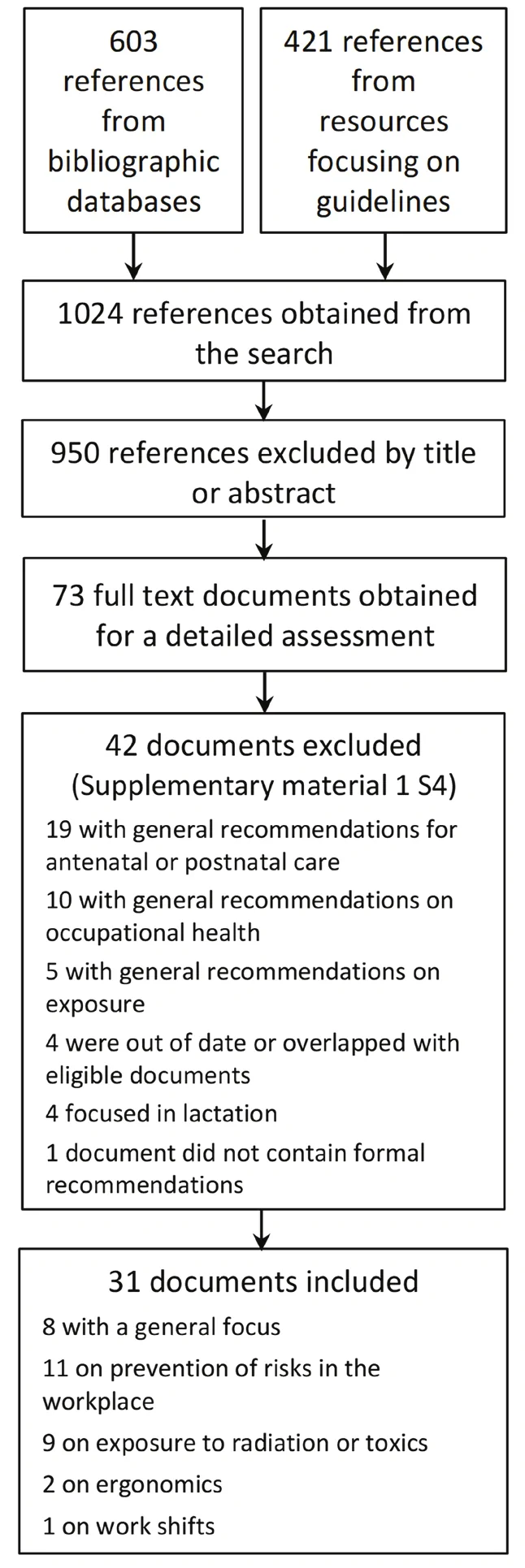
Eligibility flowchart.

The search identified 1024 references of which 603 were obtained from bibliographic databases and 421 from guideline-focused resources and 73 documents were selected for detailed assessment. After analyzing their full text, 42 documents were excluded for various reasons (supplementary material S4). After exclusions, we included 31 documents in the review (figure 1).

### Included documents

The 31 included documents (23–53) are set out in table 3. They were developed by a mixture of professional organisations and governmental institutions (including occupational medicine institutions, scientific societies) as well as a labor union and an insurance company.

**Table 3:** Characteristics of included guidelines.

ID	Institution	Type of institution	Country / region	Title	Scope
ACOEM 2016 (23)	American College of Occupational and Environmental Medicine	Professional organization	US	Reproductive and Developmental Hazard Management	Exposure to toxics
ACOG533 2016 (24)	American College of Obstetricians and Gynecologists	Professional organization	US	Lead Screening During Pregnancy and Lactation	Exposure to lead
ACOG733 2018 (25)	American College of Obstetricians and Gynecologists	Professional organization	US	Employment Considerations During Pregnancy and the Postpartum Period	General, Occupational Health
ACOG832 2021 (26)	American College of Obstetricians and Gynecologists	Professional organization	US	Reducing Prenatal Exposure to Toxic Environmental Agents	Exposure to toxics
AEEMT 2011 (27)	Asociación Española de Especialistas en Medicina del Trabajo	Scientific Society-OH**	Spain	Guía Clínico Laboral para la Prevención de Riesgos durante el Embarazo, Parto Reciente y Lactancia en el ámbito sanitario	Assessment and prevention of workplace risks
AMAT 2008 (28)	Asociación de Mutuas de Accidentes de Trabajo	Other***-OH**	Spain	Guía Médica para la Valoración de los Riesgos Profesionales a efectos de la Prestación de Riesgo durante el Embarazo y riesgo en la Lactancia	Assessment and prevention of workplace risks
ANMTAS 2008 (29)	Asociación Nacional de Medicina del Trabajo en el Ámbito Sanitario	Scientific Society-OH**	Spain	Guía de valoración de riesgos laborales en el embarazo y lactancia en trabajadoras del ámbito sanitario	Assessment and prevention of workplace risks
ASPB 2019 (30)	Agència de Salut Pública de Barcelona	Governmental	Spain	Risc laboral i prestacions per risc durant l’embaràs i la lactància natural	Assessment and prevention of workplace risks
CDC 2010 (31)	Centers for Disease Control and Prevention	Governmental	US	Guidelines for the identification and management of lead exposure in pregnant and lactating women	Exposure to lead
CSN 2016 (32)	Consejo de Seguridad Nuclear	Governmental	Spain	Protección de las trabajadoras gestantes expuestas a radiaciones ionizantes en el ámbito sanitario	Exposure to radiation
EHRA 2017 (33)	European Heart Rhythm Association	Scientific Society	Europe	Occupational radiation exposure in the electrophysiology laboratory with a focus on personnel with reproductive potential and during pregnancy	Exposure to radiation
EHRA 2023 (34)	European Heart Rhythm Association	Scientific Society	Europe	Radiation protection for healthcare professionals working in catheterisation laboratories during pregnancy	Exposure to radiation
ESVS 2023 (35)	European Society for Vascular Surgery	Scientific Society	Europe	European Society for Vascular Surgery (ESVS) 2023 Clinical Practice Guidelines on Radiation Safety	Exposure to radiation
HSE INDG334 2015 (36)	Health and Safety Executive	Governmental	UK	Working safely with ionising radiation. Guidelines for expectant or breastfeeding mothers	Exposure to radiation
HSE INDG373 2003 (37)	Health and Safety Executive	Governmental-OH**	UK	New and expectant mothers at work	General, Occupational Health
HSEweb 2023 (38)	Health and Safety Executive	Governmental-OH**	UK	Protecting pregnant workers and new mothers.	General, Occupational Health
IBV 2004 (39)	Instituto de Biomecánica de Valencia	Governmental	Spain	Requisitos ergonómicos para la protección de la maternidad en tareas con carga física	Ergonomics
ILO 2004 (40)	International Labour Organization	Governmental	Global	Healthy beginnings: Guidance on safe maternity at work.	General, Occupational Health
IMEX (41)(*)	Instituto de la Mujer de Extremadura, UGT Extremadura	Other***	Spain	Guía Práctica para trabajadoras embarazadas y en periodo de lactancia.	General, Occupational Health
INSHT/INSST 2011 (42)	Instituto Nacional de Seguridad y Salud en el Trabajo	Governmental-OH**	Spain	Directrices para la evaluación de riesgos y protección de la maternidad en el trabajo.	Assessment and prevention of workplace risks
INSS 2008 (43)	Instituto Nacional de la Seguridad Social	Governmental-OH**	Spain	Orientaciones para la valoración del riesgo laboral y la incapacidad temporal durante el embarazo.	Assessment and prevention of workplace risks
INSS 2011 (44)	Instituto Nacional de la Seguridad Social	Governmental	Spain	Guía de ayuda para la valoración de riesgo laboral durante el embarazo.	Assessment and prevention of workplace risks
ISTAS 2008 (45)	Instituto Sindical de Trabajo, Ambiente y Salud	Other***-OH**	Spain	Guía sindical para la prevención de riesgos durante el embarazo y la lactancia.	Assessment and prevention of workplace risks
ISTAS 2009 (46)	Instituto Sindical de Trabajo, Ambiente y Salud	Other***-OH**	Spain	Guía sindical para la prevención de riesgos para la reproducción, el embarazo y la lactancia.	Assessment and prevention of workplace risks
ISTAS 2013 (47)	Instituto Sindical de Trabajo, Ambiente y Salud	Other***-OH**	Spain	Guía sindical para la prevención de los riesgos durante el embarazo y la lactancia en el sector de la limpieza de edificios e instalaciones.	Assessment and prevention of workplace risks
NIOSH 1977 (48)	US National Institute for Occupational Safety and Health	Governmental-OH**	US	Guidelines on pregnancy and work.	General, Occupational Health
NIOSH 2013 (49)	US National Institute for Occupational Safety and Health	Governmental-OH**	US	Clinical guidelines for occupational lifting in pregnancy: evidence summary and provisional recommendations.	Ergonomics
OSALAN 2005 (50)	Laneko Segurtasun eta Osasunerako Euskal Erakundea	Governmental-OH**	Spain	Guía de prevención de riesgos laborales para la mujer trabajadora en situación de embarazo, que haya dado a luz o de lactancia.	Assessment and prevention of workplace risks
RCP 2009 (51)	Royal College of Physicians	Professional organization	UK	Physical and Shift Work in Pregnancy: Occupational aspects of management.	Shifts
RCP 2013 (52)	Royal College of Physicians	Professional organization	UK	Pregnancy: Occupational aspects of management.	General, Occupational Health
ID	INSTITUTION	TYPE OF INSTITUTION	COUNTRY/ REGION	TITLE	SCOPE
SOGC 2017 (53)	Society of Obstetricians and Gynaecologists of Canada	Scientific Society	Canada	Maternity Leave in Normal Pregnancy.	General, Occupational Health

* Date not reported. ** Governmental institution, scientific society or other type of institution focused on occupational health. *** Other: includes one private association of insurance companies (AMAT) and two institutions linked to the trade unions UGT (IMEX) and CCOO (ISTAS).

Although the documents covered a wide range of topics, a majority had a general focus or were centered on the assessment and prevention of risks in the workplace. Nine documents focused on the impact of exposure to radiation, lead, or toxics from a general perspective. The remaining documents evaluated ergonomic aspects or shift work.

### Quality of documents

The methodological quality of guidelines included varied considerably across the AGREE II domains (table 4).

**Table 4:** Methodological quality of included documents, according to AGREE II scores, and ranked from highest to lowest quality according to the score received at the ‘Rigor’ domain 3.

Guideline		DOMAIN I Scope		DOMAIN II Stakeholders			DOMAIN III Rigor		DOMAIN IV Clarity	DOMAIN V Applicability	DOMAIN VI Independence	Overall quality* (0–7)
RCP 2009 (51)		94.44		65.27			85.41		94.44	52.08	75.00	6
SVS 2023 (35)		86.11		87.50			71.87		88.88	35.42	56.25	6
RCP 2013 (52)		77.77		75.00			57.81		75.00	44.79	31.25	6
EHRA 2017 (33)		86.11		62.50			53.64		73.61	31.25	41.66	6
NIOSH 1977 (48)		84.72		62.50			36.97		52.77	42.70	14.58	3
NIOSH 2013 (49)		86.11		37.50			35.41		72.22	23.95	50.00	4
AEEMT 2011 (27)		70.83		50.00			32.81		63.88	35.41	4.16	4
SOGC 2017 (53)		65.27		44.44			32.29		52.77	7.29	43.75	3
INSS 20112 (44)		54.16		51.38			31.77		54.16	13.54	14.58	3
CDC 2010 (31)		80.55		54.16			29.16		55.55	33.33	16.66	3
CSN 2016 (32)		68.05		37.50			27.60		63.88	50.00	4.16	4
IBV 2004 (39)		75.00		54.16			27.08		72.22	55.20	37.50	3
ASPB 2019 (30)		44.44		23.61			26.52		66.66	33.33	4.16	3
ANMTAS 2008 (29)		75.00		56.94			21.87		62.50	35.41	4.16	3
ACOG 733 (25)		41.66		40.27			20.83		65.27	33.33	0.00	3
ACOEM 2016 (23)		56.94		38.88			20.83		44.44	31.25	64.58	2
INSS 2008 (43)		52.77		43.05			19.79		56.94	8.33	12.50	2
AMAT 2008 (28)		45.83		18.05			19.27		56.94	28.12	4.16	2
ACOG 533 (24)		54.16		13.88			17.18		66.66	16.66	12.50	2
ACOG 832 (26)		41.66		48.61			16.66		52.77	17.70	50.00	2
OSALAN 2005 (50)		51.38		18.05			16.14		26.38	16.66	12.50	1
IMEX (41)		66.66		25.00			15.62		56.94	32.29	6.25	1
EHRA 2023 (34)		77.77		69.44			15.10		34.72	22.91	35.41	1
ISTAS 2013 (47)		65.27		56.94			12.50		37.50	17.70	16.66	1
ISTAS 2008 (45)		80.55		41.66			11.97		65.27	29.16	4.16	2
INSHT/INSST 2011 (42)		79.16		23.611			11.97		43.05	14.58	29.16	1
ISTAS 2009 (46)		62.50		38.88			8.33		44.44	17.70	2.08	1
HSE INDG334 (36)		59.72		12.50			7.29		59.72	22.91	0.00	1
ILO 2004 (40)		65.27		29.16			4.68		62.50	33.33	8.33	2
HSE INDG373 (37)		51.38		22.22			4.68		45.83	29.16	0.00	1
HSE web (38)		100		33.33			0.00		55.55	2.08	0.00	1

*Score assigned by consensus among reviewers.

**Figure:**
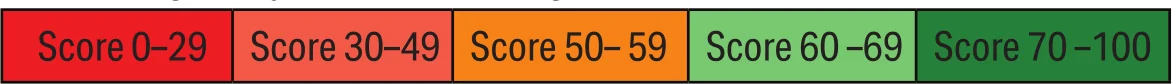

Domain III (rigor in development) was the domain with the lowest scores among domains related to methodological quality (domains III and IV). Only four guidelines achieved scores >50% (33, 35, 51, 52), indicating at least a moderate level of methodological quality in their development. Of those, only two guidelines achieved scores >70% (35, 51).

Seventy-one percent of the documents (N=22) presented important methodological limitations related to the lack (or limited description) of the methods used to formulate the recommendations, obtaining scores <30% (23–26, 28–32, 34, 36–43, 45–47, 50). The limitations most frequently identified were the lack of an explicit description of the methods used to conduct the literature search (24–26, 36–38, 40, 41, 45, 46) and a description of the methodology used to formulate the recommendations or limitations of the scientific literature used in all 22 documents. In 17 documents, the relationship between the scientific evidence and the proposed recommendations was not clearly explained (27, 28, 30, 32, 36–41, 44–46, 50, 53). Likewise, most guidelines provided little or no information on the procedures planned for future updating.

The scores for domain IV (clarity of presentation) were generally higher than those described for domain III. Twenty-four guidelines (77.4%) achieved scores ≥50%, indicating that, in general, the recommendations were presented in a clear and structured manner (24–33, 35, 36, 38–41, 43–45, 48, 49, 51–53). However, only 14 guidelines (45.2%) achieved scores >60%, demonstrating a higher level of clarity and greater ease in identifying the key recommendations (24, 25, 27, 29, 30, 32, 33, 35, 39, 40, 45, 49, 51, 52).

Despite these relatively favorable scores, some shortcomings were observed. Eight guidelines did not formulate their recommendations using specific and clear statements (23, 26, 31, 33, 37, 45, 47, 53), whereas in 11 documents the recommendations were not easily identifiable within the text (23, 31, 34, 36–38, 42, 45–48).

The scores obtained by the documents in domain I (scope and purpose) were generally higher. Only four guidelines scored <50%, mainly because they did not clearly define their objectives (25, 26, 28) or did not specify the clinical questions they intended to address (30).

Applicability (domain V) was one of the lowest-scoring domains. None of the guidelines provided comprehensive tools to facilitate the implementation of their recommendations in practice. Only three guidelines achieved scores ≥50% (32, 39, 51), mainly because they addressed certain aspects related to implementation, such as resource implications or auditing criteria, rather than providing practical implementation tools. In addition, 26 guidelines did not include criteria for monitoring or auditing the implementation of their recommendations.

Regarding the overall quality assessment, four guidelines received the highest score of six out of seven points (33, 35, 51, 52). In contrast, more than half of the guidelines included (51.6%) received overall quality scores of 1–2 (on a 7-point scale).

## Discussion

Our systematic evaluation using the AGREE II instrument of the 31 documents included in this study revealed substantial variability in their quality. Although most documents clearly stated their objectives and presented their recommendations in a clear manner, only four achieved adequate levels of methodological rigor during their development and overall quality. Except for three documents, the rest raised serious concerns about their applicability. Most guidelines achieved better results in the other domains.

The most relevant finding of this study was the generally low performance in domain III (rigor of development). This reflects the limitations in reporting description of the methods used to search for and synthesize scientific evidence, as well as the processes used to formulate recommendations and the procedures planned for updating them. These shortcomings are particularly important given the central role of domain III in assessing the methodology used to develop guidelines and formulate recommendations, as well as their transparency and reproducibility. The lack of information on these processes could undermine the scientific credibility of the recommendations, reducing the practical value of these documents as tools to support their use in clinical practice, particularly in the field of occupational health. However, the AGREE II instrument assesses the methodological quality and transparency of guidelines’ development process rather than the scientific validity or clinical effectiveness of the recommendations themselves. Consequently, guidelines with limited methodological rigor may nevertheless contain recommendations supported by robust scientific evidence, whereas methodologically well-developed guidelines may rely on outdated or limited evidence. Accordingly, low scores in domain III should be interpreted primarily as reflecting insufficient reporting or methodological transparency rather than necessarily indicating that the recommendations themselves are inappropriate. Another important limitation was the absence of regular and explicit updating procedures in many of the included guidelines. As scientific evidence and working conditions continue to evolve, guidelines that are not updated have the risk of becoming obsolete and potentially unsafe. Similar findings have been highlighted in broader methodological literature, where updating procedures were infrequently performed as poorly reported across clinical fields (54, 55).

The scores obtained for domain IV (clarity) were more encouraging. In most guidelines, the recommendations were presented in a clear and structured manner. This suggests that recommendations can appear clear despite being supported by a scarce description of their development methods. However, only about half of the guidelines allowed readers to easily identify the recommendations within the document. This suggests that greater attention was given to the wording of recommendations than to facilitating their understanding by end users, indicating that the usability of these documents in routine occupational health practice could be further improved.

These findings are consistent with several recent systematic appraisals of general pregnancy-related clinical practice guidelines using AGREE II. Systematic reviews of guidelines addressing COVID-19 management, nutrition and other areas of maternity care have consistently reported that, although many guidelines score highly in scope and purpose and clarity of presentation, the majority perform poorly in rigor of development, applicability and editorial independence (56–60). This body of evidence shows that methodological limitations in the development of guidelines are not only presented in the field of occupational health but reflect a condition in many areas of medicine and pregnancy-related clinical practice.

Scores in domain V (applicability) were generally low. The absence of practical tools to facilitate the implementation of recommendations was observed in most of the included documents. In the management of occupational risks during pregnancy, this issue is especially relevant, because implementation of the recommendations depends on collaboration among clinicians, occupational health professionals, employers, and workers. Domain II (stakeholder involvement) also received relatively low scores, indicating limited participation of experts from different disciplines in the development of the documents included. The involvement of multiple stakeholders is therefore a key element for improving methodological quality of guidelines and their applicability and acceptance by both healthcare professionals and employers.

Regarding overall quality, ratings were closely associated with domain III scores. Although AGREE II does not establish official cutoff values, the strong relationship observed between overall quality ratings and methodological rigor suggests that reviewers considered the rigor of guideline development to be a major determinant of their global assessment. This finding is consistent with the purpose of the AGREE II instrument, which emphasizes methodological quality as the foundation for trustworthy recommendations.

These findings suggest that guidelines may influence the work-life balance of pregnant workers. Decisions based on low-quality guidelines can lead to unnecessary or poorly managed absences from work, which in turn can negatively affect workers’ professional development, their income, the financial stability of their families (15) and for employers. The correct implementation of clinical practice guidelines could mitigate these negative effects (15). Besides, emerging evidence indicates that organizational support, appropriate workload, and workplace accommodations improve maternal well-being and facilitate return to work after maternity leave (1, 4, 61, 62). Consequently, future guidelines should better integrate these psychosocial aspects into occupational risk management.

Going forward, guideline development should prioritize transparent evidence synthesis, explicit grading of recommendations, multidisciplinary participation, practical implementation tools, and regular updating procedures. Together, these elements would contribute to improving the relevance, applicability, and long-term effectiveness of guidelines in occupational and public health practice (18, 54, 55).

The findings of this study should be interpreted considering several limitations. First, although the AGREE II instrument was originally developed to evaluate clinical practice guidelines, its domains and items are sufficiently generic to allow its application to guidance documents in other fields, including occupational health. The AGREE II instrument does not recommend universal cutoff values to classify guidelines according to methodological quality. Likewise, there is no validated evidence regarding the sensitivity or discriminatory performance of specific cutoff values for distinguishing between high- and low-quality guidelines. Consequently, the 50% threshold adopted in this study should be interpreted as a pragmatic criterion established a priori to facilitate descriptive comparisons among documents rather than as a validated standard of guideline quality. Four reviewers independently conducted this appraisal, three of whom had formal training in the use of the AGREE II instrument, following standardized procedures consistent with the AGREE II methodology. Nevertheless, some degree of subjectivity is inherent to this type of appraisal and cannot be completely eliminated, which may explain the variability observed in individual item scores.

Second, the project originated within the Spanish occupational health context, which may have introduced some bias in the identification of relevant documents from other countries. However, the search strategy was specifically designed to identify documents published in English, Italian, Portuguese, and Spanish, and targeted searches were conducted on the websites of relevant national and international organizations. Moreover, the methodological quality of the Spanish guidelines was comparable to that of most guidelines identified from other countries.

Third, the review was restricted to publicly available documents, and therefore internal or unpublished guidelines may not have been identified.

Finally, differences in legal frameworks, occupational health systems, and maternity protection policies across countries may limit the direct transferability of some recommendations to other settings.

Despite these limitations, this study has several important strengths. To our knowledge, it is the first systematic review to identify and critically appraise the methodological quality of guidelines specifically addressing occupational risk management during pregnancy using the AGREE II instrument. The inclusion of documents from multiple countries provides a comprehensive overview of current international practice and enhances the relevance of the findings beyond a single national context. By identifying systematic methodological weaknesses across guidelines, this review provides a foundation for improving guideline development and promoting more transparent, evidence-based recommendations to support occupational health practice for pregnant workers .

### Concluding remarks

The findings of this study emphasize the need for significant improvement of the quality of clinical practice guidelines in the field of protecting pregnant women from occupational risks. It is imperative that rigorous and standardized methodological approaches are adopted in the development of these guidelines and that their continuous updating and participation of all relevant stakeholders are ensured. Only in this way it can be guaranteed that the recommendations provided could be effective and beneficial for pregnant workers and their employers, contributing not only to risk prevention but also to the promotion of a work environment that supports the balance between work and personal life during this crucial stage.

## Supplementary material

Supplementary material
