# Supplementary material for: Quality of guidelines for the management and prevention of occupational risks during pregnancy: a systematic quality assessment using the Appraisal of Guidelines for Research and Evaluation (AGREE II)
Source: Scand J Work Environ Health. 2026 Aug 31;52(5):473–85. doi: 10.5271/sjweh.4323 (PMC13571321; doi:10.5271/sjweh.4323)
Supplement: Supplementary file 1 [file SJWEH-52-473-S001.pdf]

Quality of guidelines for the management and prevention of occupational risks during pregnancy: a systematic quality assessment using the Appraisal of Guidelines for Research and Evaluation (AGREE II)[1]

by Martina Lupia, MD, Ivan Solà, PhD, Rocío Villar, PhD, Marco Culqui, MSc, Pilar Díaz, OHN, Marilina Santero, PhD, Joan Mirabent, OHN, Fernando G Benavides, PhD, Consol Serra, PhD[2]

1. Supplementary materials
2. Correspondence to: Consol Serra, MD, MSc, PhD, CiSAL-Centre for Research in Occupational Health, Hospital del Mar Research Institute / Universitat Pompeu Fabra, PRBB Building, Dr. Aiguader, 88, 08003-Barcelona, Spain. [E-mail: consol.serra@upf.edu]

S1. Search strategies used in electronic searches to identify documents of interest 2

S2. Calculations to obtain the scores for the AGREE II domains 4

S3. AGREE's scores of included documents, raw data per domain 5

S4. Reasons for exclusion after full-text screening 14

S1. Search strategies used in electronic searches to identify documents of interest

MEDLINE
PubMed
02/11/2023

1	"Pregnancy"[Mesh]	1,015,932
2	"Pregnancy Complications"[Majr]	386,768
3	"Maternal Exposure"[Mesh]	11,186
4	reproductive[ti]	59,562
5	pregnan*[ti]	269,133
6	antenatal[ti]	12,661
7	breastfeed*[ti]	17,007
8	"breast feed*"[ti]	5,22
9	lactation[ti]	12,975
10	milk[ti]	64,229
11	matern*[ti]	107,4
12	#1 OR #2 OR #3 OR #4 OR #5 OR #6 OR #7 OR #8 OR #9 OR #10 OR #11	1,223,897
13	"Work"[Mesh]	70,679
14	"Employment"[Mesh]	100,855
15	"Occupations"[Mesh]	36,825
16	"Women, Working"[Mesh]	5,589
17	"Work Schedule Tolerance"[Mesh]	7,564
18	"Occupational Exposure"[Mesh]	69,782
19	"Occupational Health"[Mesh]	36,698
20	"Occupational Health Services"[Mesh]	10,763
21	work*[ti]	294,964
22	occupation*[ti]	64,744
23	employ*[ti]	42,315
24	#13 OR #14 OR #15 OR #16 OR #17 OR #18 OR #19 OR #20 OR #21 OR #22 OR #23	552,982
25	#24 AND #12	16,138
26	"Guideline"[pt]	37,913
27	guideline*[ti]	96,194
28	recommendation*[ti]	51,327
29	statement[ti]	19,308
30	"practice guidelines"[tiab]	29,273
31	"Guidelines as Topic"[Mesh]	172,965
32	Committee*[ti]	28,706
33	consensus[ti]	33,357
34	#26 OR #27 OR #28 OR #29 OR #30 OR #31 OR #32 OR #33	311,631
35	#25 AND #34	493

CINAHL
EBSCOHost
31/07/2023

S1	(MM "Pregnancy")	4,547
S2	(MM "Pregnancy Complications")	15,469
S3	TI reproductive OR pregnan* OR antenatal OR breastfeed* OR "breast feed*"	118,054
S4	S1 OR S2 OR S3	126,076
S5	(MH "Occupational Diseases+")	44,183
S6	(MH "Air Pollutants, Occupational")	1,495
S7	(MH "Work Environment")	39,107
S8	(MH "Work")	7,371
S9	(MH "Employment")	24,226
S10	TI work* OR occupation* OR job OR employ*	228,742
S11	S5 OR S6 OR S7 OR S8 OR S9 OR S10	290,129
S12	S4 AND S11	2,023
S13	(MH "Practice Guidelines")	85,252

S14	TI guideline* OR recommendation* OR statement	81,821
S15	S13 OR S14	139,406
S16	S12 AND S15	44

EMBASE
embase.com
31/07/2023

#1	'pregnancy'/mj	153,369
#2	'pregnancy complication'/mj	9,147
#3	reproductive:ti	47,678
#4	pregnan*:ti	250,160
#5	antenatal:ti	12,852
#6	breastfeed*:ti	13,051
#7	'breast feed*':ti	3,313
#8	#1 OR #2 OR #3 OR #4 OR #5 OR #6 OR #7	361,584
#9	'work'/de	19,997
#10	'employment'/mj	7,408
#11	'occupational health'/de	27,046
#12	work*:ti	194,202
#13	occupation*:ti	46,847
#14	employ*:ti	26,736
#15	#9 OR #10 OR #11 OR #12 OR #13 OR #14	285,041
#16	#8 AND #15	2,697
#17	'practice guideline'/mj	70,500
#18	guideline*:ti	95,649
#19	recommendation*:ti	47,907
#20	statement:ti	14,496
#21	'practice guideline*':ti,ab	36,960
#22	#17 OR #18 OR #19 OR #20 OR #21	195,732
#23	#16 AND #22	66

S2. Calculations to obtain the scores for the AGREE II domains.

DOMAIN	SCORES CALCULATION
GENERAL FORMULA	((total domain obtained score – minps[1])/(maxps[2] – minps))*100<br>maxps = 7 (strongly agree) * domain items * n appraisers<br>minps = 1 (strongly disagree) * domain items * n appraisers
Domain 1	((total domain obtained score – 12)/(84-12))*100<br>maxps = 7*3*4 = 84<br>minps = 1*3*4 = 12
Domain 2	((total domain obtained score – 12)/ (84-12))*100<br>maxps = 7*3*4 = 84<br>minps = 1*3*4 = 12
Domain 3	((total domain obtained score – 32)/(224-32))*100<br>maxps = 7*8*4 = 224<br>minps = 1*8*4 = 32
Domain 4	((total domain obtained score – 12)/(84-12))*100<br>maxps = 7*3*4 = 84<br>minps = 1*3*4 = 12
Domain 5	((total domain obtained score – 16)/(112-16))*100<br>maxps = 7*4*4 = 112<br>minps = 1*4*4 = 16
Domain 6	((total domain obtained score – 8)/(56-8)*100<br>maxps = 7*2*4 = 56<br>minps = 1*2*4 = 8

[1]minps = minimum possible score; [2]maxps = maximum possible

S3. AGREE's scores for included documents, raw data per domain (**NB** IT#Appr# in columns stand for the scores at a specific item, provided by each appraiser)

DOMAIN 1 – SCOPE AND PURPOSE (items 1 to 3) (**NB** DOMAIN SCORE calculated according to ((TOTAL – 12)/72)*100; see Table 3 for additional details)

DOCUMENT ID	IT1Appr1	IT1Appr2	IT1Appr3	IT1Appr4	IT2Appr1	IT2Appr2	IT2Appr3	IT2Appr4	IT3Appr1	IT3Appr2	IT3Appr3	IT3Appr4	TOTAL	DOMAIN SCORE
ACOEM 2016	2	7	5	5	2	6	6	5	2	5	3	5	53	56,94444444
ACOG 533	1	3	6	4	1	6	6	6	1	5	7	5	51	54,16666667
ACOG 733	1	3	2	2	1	6	5	5	1	5	6	5	42	41,66666667
ACOG 832	1	4	2	2	1	6	7	6	1	5	3	4	42	41,66666667
AEEMT 2011	4	7	6	6	2	7	5	5	2	7	6	6	63	70,83333333
AMAT 2008	2	6	2	2	1	7	3	3	2	7	5	5	45	45,83333333
ANMTAS 2008	6	7	6	6	3	7	6	6	2	7	5	5	66	75
ASPB 2019	1	1	6	1	1	5	6	5	1	5	7	5	44	44,44444444
CDC 2010	4	7	7	7	5	7	4	5	5	7	6	6	70	80,55555556
CSN 2016	1	6	7	6	1	6	6	6	1	7	7	7	61	68,05555556
EHRA 2017	5	7	7	7	4	7	5	5	6	7	7	7	74	86,11111111
EHRA 2023	2	7	6	7	2	6	7	7	3	7	7	7	68	77,77777778
ESVS 2023	6	7	7	7	6	6	4	7	5	6	6	7	74	86,11111111
HSE INDG334	1	7	6	7	1	7	2	2	1	7	7	7	55	59,72222222
HSE INDG373	1	6	5	6	1	6	2	2	1	6	7	6	49	51,38888889
HSE web	7	7	7	7	7	7	7	7	7	7	7	7	84	100
IBV 2004	3	7	7	7	2	6	7	6	2	6	7	6	66	75
ILO 2004	1	6	6	6	1	6	7	6	1	6	7	6	59	65,27777778
IMEX	1	7	7	7	1	5	7	5	1	6	7	6	60	66,66666667
INSHT/INSST 2011	6	7	7	7	5	7	1	7	3	6	7	6	69	79,16666667
INSS 2008	1	7	3	3	1	7	5	5	1	7	5	5	50	52,77777778
INSS 2009	2	6	3	3	1	6	4	3	1	6	4	3	42	41,66666667
INSS 2011	2	6	3	3	1	6	5	6	2	6	5	6	51	54,16666667
ISTAS 2008	3	7	7	7	2	7	7	7	2	7	7	7	70	80,55555556
ISTAS 2009	3	7	7	7	2	7	1	1	2	7	6	7	57	62,5
ISTAS 2013	3	7	7	7	2	7	1	2	3	7	6	7	59	65,27777778
NIOSH 1977	4	7	7	7	4	7	7	7	2	7	7	7	73	84,72222222
NIOSH 2013	5	7	7	7	4	7	7	7	2	7	7	7	74	86,11111111
OSALAN 2005	1	4	6	4	1	5	6	5	1	5	6	5	49	51,38888889
RCP 2009	6	7	7	7	6	7	7	7	5	7	7	7	80	94,44444444
RCP 2013	5	7	4	5	5	7	5	5	5	7	6	7	68	77,77777778
SOGC 2017	1	7	7	7	1	7	5	5	2	6	5	6	59	65,27777778

ACOEM American College of Occupational and Environmental Medicine; **ACOG** American College of Obstetricians and Gynecologists; **AEEMT** Asociación Española de Especialistas en Medicina del Trabajo; **AMAT** Asociación de Mutuas de Accidentes de Trabajo ; **ANMTAS** Asociación Nacional de Medicina del Trabajo en el Ámbito Sanitario, **ASPB** Agència de Salut Pública de Barcelona; **CDC** Centers for Disease Control and Prevention; **CNS** Consejo de Seguridad Nuclear; **EHRA** European Heart Rhythm Association; **ESVS** European Society for Vascular Surgery, **HSE** Health and Safety Executive; **IBV** Instituto de Biomecánica de Valencia; **ILO** International Labour Organization; **IMEX** Instituto de la Mujer de Extremadura; **INSHT/INSST** Instituto Nacional de Seguridad y Salud en el Trabajo; **INSS** Instituto Nacional de la Seguridad Social; **ISTAS** Instituto Sindical de Trabajo, Ambiente y Salud; **NIOSH** National Institute for Occupational Safety and Health; **OSALAN** Laneko Segurtasun eta Osasunerako Euskal Erakundea; **RCP** Royal College of Physicians; **SOGC** Society of Obstetricians and Gynaecologists of Canada

DOMAIN 2 – STAKEHOLDER INVOLVEMENT (items 4 to 6) (**NB** DOMAIN SCORE calculated according to ((TOTAL – 12)/72)*100; see Table 3 for additional details)

DOCUMENT ID	IT4Appr1	IT4Appr2	IT4Appr3	IT4Appr4	IT5Appr1	IT5Appr2	IT5Appr3	IT5Appr4	IT6Appr1	IT6Appr2	IT6Appr3	IT6Appr4	TOTAL	DOMAIN SCORE
ACOEM 2016	2	4	6	4	1	1	3	1	2	6	5	5	40	38,88888889
ACOG 533	1	2	4	2	1	1	1	1	1	4	2	2	22	13,88888889
ACOG 733	1	5	7	5	1	1	5	1	1	4	6	4	41	40,27777778
ACOG 832	2	5	7	5	1	4	3	3	2	5	5	5	47	48,61111111
AEEMT 2011	4	6	6	6	1	1	3	1	4	5	6	5	48	50
AMAT 2008	1	1	2	1	1	1	2	1	2	4	5	4	25	18,05555556
ANMTAS 2008	6	6	3	6	1	1	3	1	7	7	5	7	53	56,94444444
ASPB 2019	2	3	5	3	1	1	3	1	1	1	7	1	29	23,61111111
CDC 2010	6	7	6	6	1	1	1	1	4	6	6	6	51	54,16666667
CSN 2016	2	3	4	3	1	1	4	1	1	6	7	6	39	37,5
EHRA 2017	6	7	7	7	1	5	5	5	2	6	3	3	57	62,5
EHRA 2023	6	7	7	7	1	5	6	5	2	6	5	5	62	69,44444444
ESVS 2023	7	7	7	7	6	4	7	3	7	7	6	7	75	87,5
HSE INDG334	1	2	1	1	1	1	1	1	1	7	2	2	21	12,5
HSE INDG373	1	2	1	1	1	1	1	1	1	7	5	6	28	22,22222222
HSE web	1	1	1	1	1	1	1	1	7	7	7	7	36	33,33333333
IBV 2004	2	5	7	6	1	1	7	1	2	6	7	6	51	54,16666667
ILO 2004	1	6	1	1	1	1	1	1	1	7	6	6	33	29,16666667
IMEX	1	1	2	1	1	1	2	1	1	6	7	6	30	25
INSHT/INSST 2011	1	3	1	1	1	1	1	1	4	7	4	4	29	23,61111111
INSS 2008	2	7	5	5	1	1	3	1	1	7	5	5	43	43,05555556
INSS 2009	1	3	3	3	1	1	3	1	1	1	5	1	24	16,66666667
INSS 2011	5	7	5	5	1	1	3	1	4	7	5	5	49	51,38888889
ISTAS 2008	2	6	2	2	1	4	1	1	2	7	7	7	42	41,66666667
ISTAS 2009	2	6	1	2	1	4	1	1	2	7	6	7	40	38,88888889
ISTAS 2013	1	7	1	1	1	7	5	7	3	7	6	7	53	56,94444444
NIOSH 1977	2	7	7	7	1	1	7	1	3	7	7	7	57	62,5
NIOSH 2013	2	6	1	2	1	1	1	1	3	7	7	7	39	37,5
OSALAN 2005	1	3	6	1	1	1	2	1	1	2	5	1	25	18,05555556
RCP 2009	4	7	7	7	1	1	5	1	5	7	7	7	59	65,27777778
RCP 2013	6	7	6	6	1	7	4	4	6	7	6	6	66	75
SOGC 2017	2	7	4	4	1	1	1	1	2	7	7	7	44	44,44444444

ACOEM American College of Occupational and Environmental Medicine; **ACOG** American College of Obstetricians and Gynecologists; **AEEMT** Asociación Española de Especialistas en Medicina del Trabajo; **AMAT** Asociación de Mutuas de Accidentes de Trabajo ; **ANMTAS** Asociación Nacional de Medicina del Trabajo en el Ámbito Sanitario, **ASPB** Agència de Salut Pública de Barcelona; **CDC** Centers for Disease Control and Prevention; **CNS** Consejo de Seguridad Nuclear; **EHRA** European Heart Rhythm Association; **ESVS** European Society for Vascular Surgery, **HSE** Health and Safety Executive; **IBV** Instituto de Biomecánica de Valencia; **ILO** International Labour Organization; **IMEX** Instituto de la Mujer de Extremadura; **INSHT/INSST** Instituto Nacional de Seguridad y Salud en el Trabajo; **INSS** Instituto Nacional de la Seguridad Social; **ISTAS** Instituto Sindical de Trabajo, Ambiente y Salud; **NIOSH** National Institute for Occupational Safety and Health; **OSALAN** Laneko Segurtasun eta Osasunerako Euskal Erakundea; **RCP** Royal College of Physicians; **SOGC** Society of Obstetricians and Gynaecologists of Canada

DOMAIN 3 – RIGOUR OF DEVELOPMENT (items 7 to 14) (**NB** DOMAIN SCORE calculated according to ((TOTAL – 32)/192)*100; see Table 3 for additional details)

DOCUMENT ID	IT7Appr1	IT7Appr2	IT7Appr3	IT7Appr4	IT8Appr1	IT8Appr2	IT8Appr3	IT8Appr4	IT9Appr1	IT9Appr2	IT9Appr3	IT9Appr4
ACOEM 2016	1	1	6	1	1	1	2	1	1	1	1	1
ACOG 533	1	1	1	1	1	1	2	1	1	3	3	3
ACOG 733	1	1	3	1	1	1	2	1	1	3	3	3
ACOG 832	1	1	3	1	1	1	1	1	1	5	1	1
AEEMT 2011	1	1	5	1	1	1	5	1	1	4	5	4
AMAT 2008	1	1	5	1	1	1	4	1	1	1	4	1
ANMTAS 2008	1	1	4	1	1	1	4	1	1	4	3	3
ASPB 2019	1	1	7	1	1	1	7	1	1	2	5	1
CDC 2010	1	6	2	3	1	5	1	1	1	6	2	2
CSN 2016	1	1	5	1	1	1	6	1	1	1	5	1
EHRA 2017	2	7	6	6	2	7	2	2	1	7	4	4
EHRA 2023	1	3	4	3	1	1	1	1	1	1	3	1
ESVS 2023	6	7	7	7	5	6	7	6	2	4	7	4
HSE INDG334	1	1	1	1	1	1	1	1	1	1	1	1
HSE INDG373	1	1	1	1	1	1	1	1	1	1	1	1
HSE web	1	1	1	1	1	1	1	1	1	1	1	1
IBV 2004	1	1	7	1	1	1	7	1	1	1	7	1
ILO 2004	1	1	1	1	1	1	1	1	1	1	1	1
IMEX	1	1	3	1	1	1	4	1	1	1	5	1
INSHT/INSST 2011	1	7	1	1	1	1	1	1	1	1	1	1
INSS 2008	1	1	5	1	1	1	5	1	1	1	4	1
INSS 2009	1	4	4	4	1	1	4	1	1	1	3	1
INSS 2011	1	5	4	5	1	3	4	3	1	1	3	1
ISTAS 2008	1	1	1	1	1	1	1	1	1	1	1	1
ISTAS 2009	1	1	1	1	1	1	1	1	1	1	1	1
ISTAS 2013	1	5	1	1	1	5	1	1	1	1	1	1
NIOSH 1977	1	5	7	5	1	1	4	1	1	1	3	1
NIOSH 2013	5	7	5	5	1	1	5	1	1	5	4	5
OSALAN 2005	1	2	6	1	1	2	5	1	1	1	4	1
RCP 2009	7	7	7	7	6	7	7	7	5	7	7	7
RCP 2013	4	7	7	7	5	4	5	5	4	7	7	7
SOGC 2017	4	7	7	7	1	7	3	3	1	1	4	1

ACOEM American College of Occupational and Environmental Medicine; **ACOG** American College of Obstetricians and Gynecologists; **AEEMT** Asociación Española de Especialistas en Medicina del Trabajo; **AMAT** Asociación de Mutuas de Accidentes de Trabajo ; **ANMTAS** Asociación Nacional de Medicina del Trabajo en el Ámbito Sanitario, **ASPB** Agència de Salut Pública de Barcelona; **CDC** Centers for Disease Control and Prevention; **CNS** Consejo de Seguridad Nuclear; **EHRA** European Heart Rhythm Association; **ESVS** European Society for Vascular Surgery, **HSE** Health and Safety Executive; **IBV** Instituto de Biomecánica de Valencia; **ILO** International Labour Organization; **IMEX** Instituto de la Mujer de Extremadura; **INSHT/INSST** Instituto Nacional de Seguridad y Salud en el Trabajo; **INSS** Instituto Nacional de la Seguridad Social; **ISTAS** Instituto Sindical de Trabajo, Ambiente y Salud; **NIOSH** National Institute for Occupational Safety and Health; **OSALAN** Laneko Segurtasun eta Osasunerako Euskal Erakundea; **RCP** Royal College of Physicians; **SOGC** Society of Obstetricians and Gynaecologists of Canada

DOMAIN 3 – RIGOUR OF DEVELOPMENT (items 7 to 14, continued)

DOCUMENT ID	IT10Appr1	IT10Appr2	IT10Appr3	IT10Appr4	IT11Appr1	IT11Appr2	IT11Appr3	IT11Appr4	IT12Appr1	IT12Appr2	IT12Appr3	IT12Appr4
ACOEM 2016	1	1	3	1	1	3	3	2	1	5	3	4
ACOG 533	1	1	1	1	1	5	7	5	2	5	4	4
ACOG 733	1	1	3	1	1	6	4	4	1	4	4	4
ACOG 832	1	1	1	1	1	6	3	4	1	4	6	4
AEEMT 2011	1	1	5	1	1	5	6	5	1	1	6	1
AMAT 2008	1	1	5	1	1	4	5	4	1	2	6	2
ANMTAS 2008	1	1	5	1	2	6	5	5	1	4	4	4
ASPB 2019	1	1	7	1	1	6	7	6	1	1	7	1
CDC 2010	1	5	2	2	4	6	2	4	5	5	1	5
CSN 2016	1	1	6	1	1	6	6	6	1	3	6	3
EHRA 2017	4	7	5	5	2	7	7	7	2	7	6	6
EHRA 2023	1	1	1	1	2	6	4	4	1	5	3	3
ESVS 2023	5	5	5	6	5	5	4	7	6	6	7	5
HSE INDG334	1	1	1	1	1	6	5	6	1	1	1	1
HSE INDG373	1	1	1	1	1	4	4	4	1	1	1	1
HSE web	1	1	1	1	1	1	1	1	1	1	1	1
IBV 2004	1	1	7	1	1	4	7	4	1	1	7	1
ILO 2004	1	1	1	1	1	4	4	4	1	1	1	1
IMEX	1	1	5	1	1	4	5	5	1	1	5	1
INSHT/INSST 2011	1	6	1	1	2	5	1	5	1	4	1	1
INSS 2008	1	1	5	1	1	5	6	5	1	3	5	3
INSS 2009	1	1	4	1	1	5	5	5	1	3	4	3
INSS 2011	1	4	5	4	1	5	5	5	1	3	5	3
ISTAS 2008	1	1	1	1	1	5	7	5	1	3	1	1
ISTAS 2009	1	1	1	1	1	5	4	5	1	3	1	1
ISTAS 2013	1	5	1	1	1	5	2	1	1	6	1	1
NIOSH 1977	1	5	2	1	2	5	7	5	1	4	6	4
NIOSH 2013	1	5	2	1	2	6	6	6	1	6	3	1
OSALAN 2005	1	2	5	1	1	2	5	1	1	2	5	1
RCP 2009	6	7	6	6	5	7	6	6	7	7	7	7
RCP 2013	2	5	5	5	3	7	7	7	5	7	6	7
SOGC 2017	1	1	1	1	1	6	2	1	1	3	1	1

ACOEM American College of Occupational and Environmental Medicine; **ACOG** American College of Obstetricians and Gynecologists; **AEEMT** Asociación Española de Especialistas en Medicina del Trabajo; **AMAT** Asociación de Mutuas de Accidentes de Trabajo ; **ANMTAS** Asociación Nacional de Medicina del Trabajo en el Ámbito Sanitario, **ASPB** Agència de Salut Pública de Barcelona; **CDC** Centers for Disease Control and Prevention; **CNS** Consejo de Seguridad Nuclear; **EHRA** European Heart Rhythm Association; **ESVS** European Society for Vascular Surgery, **HSE** Health and Safety Executive; **IBV** Instituto de Biomecánica de Valencia; **ILO** International Labour Organization; **IMEX** Instituto de la Mujer de Extremadura; **INSHT/INSST** Instituto Nacional de Seguridad y Salud en el Trabajo; **INSS** Instituto Nacional de la Seguridad Social; **ISTAS** Instituto Sindical de Trabajo, Ambiente y Salud; **NIOSH** National Institute for Occupational Safety and Health; **OSALAN** Laneko Segurtasun eta Osasunerako Euskal Erakundea; **RCP** Royal College of Physicians; **SOGC** Society of Obstetricians and Gynaecologists of Canada

DOMAIN 3 – RIGOUR OF DEVELOPMENT (items 7 to 14, continued)

DOCUMENT ID	IT13Appr1	IT13Appr2	IT13Appr3	IT13Appr4	IT14Appr1	IT14Appr2	IT14Appr3	IT14Appr4	TOTAL	DOMAIN SCORE
ACOEM 2016	1	7	7	7	1	1	1	1	72	20,83333333
ACOG 533	1	1	1	1	1	2	1	1	65	17,1875
ACOG 733	1	2	1	1	1	5	3	3	72	20,83333333
ACOG 832	1	2	1	1	1	5	1	1	64	16,66666667
AEEMT 2011	2	7	6	7	1	4	2	2	95	32,8125
AMAT 2008	1	1	2	1	1	4	2	2	69	19,27083333
ANMTAS 2008	1	1	2	1	1	1	2	1	74	21,875
ASPB 2019	1	1	4	1	1	1	4	1	83	26,5625
CDC 2010	1	5	1	1	1	4	1	1	88	29,16666667
CSN 2016	1	1	3	1	1	4	4	4	85	27,60416667
EHRA 2017	2	7	1	2	1	1	5	1	135	53,64583333
EHRA 2023	1	1	1	1	1	1	1	1	61	15,10416667
ESVS 2023	5	6	7	7	1	3	1	6	170	71,875
HSE INDG334	1	1	1	1	1	1	1	1	46	7,291666667
HSE INDG373	1	1	1	1	1	1	1	1	41	4,6875
HSE web	1	1	1	1	1	1	1	1	32	0
IBV 2004	1	1	7	1	1	1	5	1	84	27,08333333
ILO 2004	1	1	1	1	1	1	1	1	41	4,6875
IMEX	1	1	2	1	1	1	2	1	62	15,625
INSHT/INSST 2011	1	1	1	1	1	1	1	1	55	11,97916667
INSS 2008	1	1	2	1	1	1	2	1	70	19,79166667
INSS 2009	1	1	2	1	1	1	2	1	70	19,79166667
INSS 2011	1	4	5	4	1	1	2	1	93	31,77083333
ISTAS 2008	1	1	4	1	1	1	5	1	55	11,97916667
ISTAS 2009	1	1	1	1	1	4	1	1	48	8,333333333
ISTAS 2013	1	3	1	1	1	1	1	1	56	12,5
NIOSH 1977	1	3	6	3	1	7	4	4	103	36,97916667
NIOSH 2013	1	6	1	1	1	3	1	1	100	35,41666667
OSALAN 2005	1	1	2	1	1	1	2	1	63	16,14583333
RCP 2009	2	7	6	7	1	5	5	5	196	85,41666667
RCP 2013	1	1	1	1	1	1	1	1	143	57,8125
SOGC 2017	1	7	7	7	1	3	1	1	94	32,29166667

ACOEM American College of Occupational and Environmental Medicine; **ACOG** American College of Obstetricians and Gynecologists; **AEEMT** Asociación Española de Especialistas en Medicina del Trabajo; **AMAT** Asociación de Mutuas de Accidentes de Trabajo ; **ANMTAS** Asociación Nacional de Medicina del Trabajo en el Ámbito Sanitario, **ASPB** Agència de Salut Pública de Barcelona; **CDC** Centers for Disease Control and Prevention; **CNS** Consejo de Seguridad Nuclear; **EHRA** European Heart Rhythm Association; **ESVS** European Society for Vascular Surgery, **HSE** Health and Safety Executive; **IBV** Instituto de Biomecánica de Valencia; **ILO** International Labour Organization; **IMEX** Instituto de la Mujer de Extremadura; **INSHT/INSST** Instituto Nacional de Seguridad y Salud en el Trabajo; **INSS** Instituto Nacional de la Seguridad Social; **ISTAS** Instituto Sindical de Trabajo, Ambiente y Salud; **NIOSH** National Institute for Occupational Safety and Health; **OSALAN** Laneko Segurtasun eta Osasunerako Euskal Erakundea; **RCP** Royal College of Physicians; **SOGC** Society of Obstetricians and Gynaecologists of Canada

DOMAIN 4 – CLARITY OF PRESENTATION (items 15 to 17) (**NB** DOMAIN SCORE calculated according to ((TOTAL – 12)/72)*100; see Table 3 for additional details)

DOCUMENT ID	IT15Appr1	IT15Appr2	IT15Appr3	IT15Appr4	IT16Appr1	IT16Appr2	IT16Appr3	IT16Appr4	IT17Appr1	IT17Appr2	IT17Appr3	IT17Appr4	TOTAL	DOMAIN SCORE
ACOEM 2016	1	6	4	4	1	6	4	4	1	6	3	4	44	44,44444444
ACOG 533	2	6	6	5	2	6	6	5	2	7	7	6	60	66,66666667
ACOG 733	6	6	6	6	1	6	4	4	1	7	6	6	59	65,27777778
ACOG 832	1	6	3	4	1	6	5	5	2	7	5	5	50	52,77777778
AEEMT 2011	2	6	5	6	2	6	5	6	2	7	5	6	58	63,88888889
AMAT 2008	2	6	5	5	2	6	5	5	1	6	5	5	53	56,94444444
ANMTAS 2008	3	6	5	5	4	6	4	5	3	6	5	5	57	62,5
ASPB 2019	1	6	6	6	2	6	7	6	1	6	7	6	60	66,66666667
CDC 2010	4	7	4	4	3	7	1	4	4	7	3	4	52	55,55555556
CSN 2016	2	6	6	6	1	6	6	6	1	6	6	6	58	63,88888889
EHRA 2017	2	7	6	6	1	7	6	6	3	7	7	7	65	73,61111111
EHRA 2023	2	6	2	2	1	6	3	2	2	7	2	2	37	34,72222222
ESVS 2023	6	6	6	7	6	6	7	5	6	7	7	7	76	88,88888889
HSE INDG334	1	6	6	6	1	6	6	6	1	7	4	5	55	59,72222222
HSE INDG373	1	6	4	4	1	6	4	4	1	7	3	4	45	45,83333333
HSE web	2	7	2	7	2	7	2	7	1	7	1	7	52	55,55555556
IBV 2004	2	6	7	6	1	6	7	6	4	6	7	6	64	72,22222222
ILO 2004	1	6	6	6	1	6	6	6	1	6	6	6	57	62,5
IMEX	1	6	4	6	1	6	4	6	1	6	6	6	53	56,94444444
INSHT/INSST 2011	2	6	5	5	1	6	1	1	1	6	4	5	43	43,05555556
INSS 2008	2	6	5	6	1	6	5	6	1	5	5	5	53	56,94444444
INSS 2009	2	5	5	5	1	5	5	5	1	5	4	5	48	50
INSS 2011	1	5	6	5	1	5	6	5	1	5	6	5	51	54,16666667
ISTAS 2008	2	6	7	6	2	7	6	6	2	7	4	4	59	65,27777778
ISTAS 2009	3	6	2	3	2	7	4	2	2	7	4	2	44	44,44444444
ISTAS 2013	3	6	1	3	2	7	4	2	1	7	2	1	39	37,5
NIOSH 1977	3	6	5	6	1	6	5	6	1	7	3	1	50	52,77777778
NIOSH 2013	4	6	5	5	1	7	6	7	2	7	7	7	64	72,22222222
OSALAN 2005	1	2	5	4	1	2	5	2	1	2	5	1	31	26,38888889
RCP 2009	6	7	7	7	5	7	7	7	6	7	7	7	80	94,44444444
RCP 2013	4	7	6	7	3	7	3	3	5	7	7	7	66	75
SOGC 2017	2	7	4	2	1	7	4	1	2	7	6	7	50	52,77777778

ACOEM American College of Occupational and Environmental Medicine; **ACOG** American College of Obstetricians and Gynecologists; **AEEMT** Asociación Española de Especialistas en Medicina del Trabajo; **AMAT** Asociación de Mutuas de Accidentes de Trabajo ; **ANMTAS** Asociación Nacional de Medicina del Trabajo en el Ámbito Sanitario, **ASPB** Agència de Salut Pública de Barcelona; **CDC** Centers for Disease Control and Prevention; **CNS** Consejo de Seguridad Nuclear; **EHRA** European Heart Rhythm Association; **ESVS** European Society for Vascular Surgery, **HSE** Health and Safety Executive; **IBV** Instituto de Biomecánica de Valencia; **ILO** International Labour Organization; **IMEX** Instituto de la Mujer de Extremadura; **INSHT/INSST** Instituto Nacional de Seguridad y Salud en el Trabajo; **INSS** Instituto Nacional de la Seguridad Social; **ISTAS** Instituto Sindical de Trabajo, Ambiente y Salud; **NIOSH** National Institute for Occupational Safety and Health; **OSALAN** Laneko Segurtasun eta Osasunerako Euskal Erakundea; **RCP** Royal College of Physicians; **SOGC** Society of Obstetricians and Gynaecologists of Canada

DOMAIN 5 – APPLICABILITY (items 18 to 21) (**NB** DOMAIN SCORE calculated according to ((TOTAL – 16)/96)*100; see Table 3 for additional details)

DOCUMENT ID	IT18Appr1	IT18Appr2	IT18Appr3	IT18Appr4	IT19Appr1	IT19Appr2	IT19Appr3	IT19Appr4
ACOEM 2016	1	6	2	4	1	6	4	4
ACOG 533	1	3	1	1	1	3	5	3
ACOG 733	1	6	3	4	1	7	4	4
ACOG 832	1	6	2	2	1	7	1	1
AEEMT 2011	1	6	5	5	1	7	4	4
AMAT 2008	1	4	4	4	1	5	4	4
ANMTAS 2008	1	4	4	4	1	7	5	5
ASPB 2019	1	4	6	4	1	4	7	4
CDC 2010	1	6	1	1	1	7	5	5
CSN 2016	1	6	6	6	1	6	7	6
EHRA 2017	1	6	2	2	1	6	7	6
EHRA 2023	1	6	1	1	1	7	4	4
ESVS 2023	1	3	2	5	5	5	6	5
HSE INDG334	1	6	1	1	1	6	5	6
HSE INDG373	1	5	5	5	1	5	5	5
HSE web	1	1	1	1	2	1	2	1
IBV 2004	1	5	7	6	1	6	7	6
ILO 2004	1	5	3	4	1	7	5	7
IMEX	1	4	6	4	1	5	6	5
INSHT/INSST 2011	1	4	2	4	1	5	2	2
INSS 2008	1	1	4	1	1	1	3	1
INSS 2009	1	1	3	1	1	1	3	1
INSS 2011	1	1	5	1	1	1	6	1
ISTAS 2008	1	6	1	1	1	7	6	6
ISTAS 2009	1	6	1	1	1	7	1	1
ISTAS 2013	1	6	1	1	1	7	1	1
NIOSH 1977	1	6	3	1	1	6	6	6
NIOSH 2013	1	6	1	1	1	6	5	6
OSALAN 2005	1	2	5	1	1	2	5	1
RCP 2009	1	6	4	5	1	7	6	7
RCP 2013	2	7	4	4	1	7	6	7
SOGC 2017	1	4	1	1	1	4	2	1

ACOEM American College of Occupational and Environmental Medicine; **ACOG** American College of Obstetricians and Gynecologists; **AEEMT** Asociación Española de Especialistas en Medicina del Trabajo; **AMAT** Asociación de Mutuas de Accidentes de Trabajo ; **ANMTAS** Asociación Nacional de Medicina del Trabajo en el Ámbito Sanitario, **ASPB** Agència de Salut Pública de Barcelona; **CDC** Centers for Disease Control and Prevention; **CNS** Consejo de Seguridad Nuclear; **EHRA** European Heart Rhythm Association; **ESVS** European Society for Vascular Surgery, **HSE** Health and Safety Executive; **IBV** Instituto de Biomecánica de Valencia; **ILO** International Labour Organization; **IMEX** Instituto de la Mujer de Extremadura; **INSHT/INSST** Instituto Nacional de Seguridad y Salud en el Trabajo; **INSS** Instituto Nacional de la Seguridad Social; **ISTAS** Instituto Sindical de Trabajo, Ambiente y Salud; **NIOSH** National Institute for Occupational Safety and Health; **OSALAN** Laneko Segurtasun eta Osasunerako Euskal Erakundea; **RCP** Royal College of Physicians; **SOGC** Society of Obstetricians and Gynaecologists of Canada

DOMAIN 5 – APPLICABILITY (items 18 to 21, continued)

DOCUMENT ID	IT20Appr1	IT20Appr2	IT20Appr3	IT20Appr4	IT21Appr1	IT21Appr2	IT21Appr3	IT21Appr4	TOTAL	DOMAIN SCORE
ACOEM 2016	1	3	3	3	1	5	1	1	46	31,25
ACOG 533	1	1	1	1	1	5	1	3	32	16,66666667
ACOG 733	1	4	2	3	1	5	1	1	48	33,33333333
ACOG 832	1	1	2	1	1	4	1	1	33	17,70833333
AEEMT 2011	1	1	4	1	1	5	2	2	50	35,41666667
AMAT 2008	1	1	4	1	1	4	2	2	43	28,125
ANMTAS 2008	1	1	5	1	1	7	2	1	50	35,41666667
ASPB 2019	1	1	6	1	1	1	5	1	48	33,33333333
CDC 2010	1	6	2	2	1	7	1	1	48	33,33333333
CSN 2016	1	1	6	1	1	5	5	5	64	50
EHRA 2017	1	1	3	1	1	6	1	1	46	31,25
EHRA 2023	1	1	1	1	1	6	1	1	38	22,91666667
ESVS 2023	1	3	6	1	1	2	3	1	50	35,41666667
HSE INDG334	1	1	1	1	1	1	4	1	38	22,91666667
HSE INDG373	1	1	5	1	1	1	1	1	44	29,16666667
HSE web	1	1	1	1	1	1	1	1	18	2,083333333
IBV 2004	1	1	7	1	1	6	7	6	69	55,20833333
ILO 2004	1	1	2	1	1	7	1	1	48	33,33333333
IMEX	1	1	2	1	1	5	3	1	47	32,29166667
INSHT/INSST 2011	1	1	2	1	1	1	1	1	30	14,58333333
INSS 2008	1	1	3	1	1	1	2	1	24	8,333333333
INSS 2009	1	1	3	1	1	1	2	1	23	7,291666667
INSS 2011	1	1	4	1	1	1	2	1	29	13,54166667
ISTAS 2008	1	1	3	1	1	6	1	1	44	29,16666667
ISTAS 2009	1	1	2	1	1	6	1	1	33	17,70833333
ISTAS 2013	1	1	2	1	1	6	1	1	33	17,70833333
NIOSH 1977	1	1	3	1	1	7	6	7	57	42,70833333
NIOSH 2013	1	1	1	1	1	5	1	1	39	23,95833333
OSALAN 2005	1	2	5	1	1	1	2	1	32	16,66666667
RCP 2009	1	1	4	1	1	7	7	7	66	52,08333333
RCP 2013	1	1	6	1	1	5	3	3	59	44,79166667
SOGC 2017	1	1	1	1	1	1	1	1	23	7,291666667

ACOEM American College of Occupational and Environmental Medicine; **ACOG** American College of Obstetricians and Gynecologists; **AEEMT** Asociación Española de Especialistas en Medicina del Trabajo; **AMAT** Asociación de Mutuas de Accidentes de Trabajo ; **ANMTAS** Asociación Nacional de Medicina del Trabajo en el Ámbito Sanitario, **ASPB** Agència de Salut Pública de Barcelona; **CDC** Centers for Disease Control and Prevention; **CNS** Consejo de Seguridad Nuclear; **EHRA** European Heart Rhythm Association; **ESVS** European Society for Vascular Surgery, **HSE** Health and Safety Executive; **IBV** Instituto de Biomecánica de Valencia; **ILO** International Labour Organization; **IMEX** Instituto de la Mujer de Extremadura; **INSHT/INSST** Instituto Nacional de Seguridad y Salud en el Trabajo; **INSS** Instituto Nacional de la Seguridad Social; **ISTAS** Instituto Sindical de Trabajo, Ambiente y Salud; **NIOSH** National Institute for Occupational Safety and Health; **OSALAN** Laneko Segurtasun eta Osasunerako Euskal Erakundea; **RCP** Royal College of Physicians; **SOGC** Society of Obstetricians and Gynaecologists of Canada

DOMAIN 6 – EDITORIAL INDEPENDENCE (items 22 and 23) (**NB** DOMAIN SCORE calculated according to ((TOTAL – 8)/48)*100; see Table 3 for additional details)

**DOCUMENT ID**	**IT22Appr1**	**IT22Appr2**	**IT22Appr3**	**IT22Appr4**	**IT23Appr1**	**IT23Appr2**	**IT23Appr3**	**IT23Appr4**	**TOTAL**	**DOMAIN SCORE**
ACOEM 2016	1	7	1	7	2	7	7	7	39	64,58333333
ACOG 533	1	1	7	1	1	1	1	1	14	12,5
ACOG 733	1	1	1	1	1	1	1	1	8	0
ACOG 832	1	7	1	1	1	7	7	7	32	50
AEEMT 2011	1	1	2	1	1	1	2	1	10	4,166666667
AMAT 2008	1	1	2	1	1	1	2	1	10	4,166666667
ANMTAS 2008	1	1	2	1	1	1	2	1	10	4,166666667
ASPB 2019	1	1	2	1	1	1	2	1	10	4,166666667
CDC 2010	3	4	1	4	1	1	1	1	16	16,66666667
CSN 2016	1	1	2	1	1	1	2	1	10	4,166666667
EHRA 2017	4	7	1	5	2	7	1	1	28	41,66666667
EHRA 2023	1	1	6	1	4	7	1	4	25	35,41666667
ESVS 2023	1	4	4	7	2	5	5	7	35	56,25
HSE INDG334	1	1	1	1	1	1	1	1	8	0
HSE INDG373	1	1	1	1	1	1	1	1	8	0
HSE web	1	1	1	1	1	1	1	1	8	0
IBV 2004	3	5	7	5	1	1	3	1	26	37,5
ILO 2004	1	5	1	1	1	1	1	1	12	8,333333333
IMEX	1	1	3	1	1	1	2	1	11	6,25
INSHT/INSST 2011	1	5	7	5	1	1	1	1	22	29,16666667
INSS 2008	1	5	2	1	1	1	2	1	14	12,5
INSS 2009	1	5	2	2	1	1	2	1	15	14,58333333
INSS 2011	1	5	2	2	1	1	2	1	15	14,58333333
ISTAS 2008	1	3	1	1	1	1	1	1	10	4,166666667
ISTAS 2009	0	3	1	1	1	1	1	1	9	2,083333333
ISTAS 2013	2	7	1	2	1	1	1	1	16	16,66666667
NIOSH 1977	1	4	1	1	1	5	1	1	15	14,58333333
NIOSH 2013	1	6	1	1	2	7	7	7	32	50
OSALAN 2005	1	2	5	1	1	1	2	1	14	12,5
RCP 2009	1	6	7	7	2	7	7	7	44	75
RCP 2013	4	7	4	4	1	1	1	1	23	31,25
SOGC 2017	1	5	7	5	1	7	2	1	29	43,75

ACOEM American College of Occupational and Environmental Medicine; **ACOG** American College of Obstetricians and Gynecologists; **AEEMT** Asociación Española de Especialistas en Medicina del Trabajo; **AMAT** Asociación de Mutuas de Accidentes de Trabajo ; **ANMTAS** Asociación Nacional de Medicina del Trabajo en el Ámbito Sanitario, **ASPB** Agència de Salut Pública de Barcelona; **CDC** Centers for Disease Control and Prevention; **CNS** Consejo de Seguridad Nuclear; **EHRA** European Heart Rhythm Association; **ESVS** European Society for Vascular Surgery, **HSE** Health and Safety Executive; **IBV** Instituto de Biomecánica de Valencia; **ILO** International Labour Organization; **IMEX** Instituto de la Mujer de Extremadura; **INSHT/INSST** Instituto Nacional de Seguridad y Salud en el Trabajo; **INSS** Instituto Nacional de la Seguridad Social; **ISTAS** Instituto Sindical de Trabajo, Ambiente y Salud; **NIOSH** National Institute for Occupational Safety and Health; **OSALAN** Laneko Segurtasun eta Osasunerako Euskal Erakundea; **RCP** Royal College of Physicians; **SOGC** Society of Obstetricians and Gynaecologists of Canada

S4. Reasons for exclusion of after full-text screening

ID	INSTITUTION/REGION/YEAR of PUBLICATION	REASON FOR EXCLUSION	TITLE/SCOPE
**REASON FOR EXCLUSION: out of scope – general recommendations for antenatal or postnatal care**			
USPSTF 2016	US Preventive Services Task Force (USPSTF)<br>US – 2016	out of scope	Primary Care Interventions to Support Breastfeeding; Updated Systematic Review for the U.S. Preventive Services Task Force. Evidence Syntheses, No. 143
Gencat 2018	Departament de Salut, Secretaria de Salut Pública<br>Catalunya – 2018	out of scope	Guia per a embarassades
COLCIENCIAS 2013	Ministerio de Salud y Protección Social – Colciencias<br>Colombia – 2013	out of scope	Guía de Práctica Clínica para la prevención, detección temprana y tratamiento del embarazo, parto o puerperio
Osakidetza 2017	Osakidetza<br>Euskadi – 2017	out of scope	Guía del EMBARAZO, preconcepción, parto y puerperio SALUDABLE
GOIB 2017	Direcció General de Salud Pública i Participació, Servei de Salut de les Illes Balears<br>Illes Balears – 2017	out of scope	Guía de buenas prácticas en la atención del embarazo, del puerperio y del periodo neonatal en el área de salud materno infantil de las Islas Baleares
SSPA 2017	Servicio de Salud del Principado de Asturias<br>Asturias – 2017	out of scope	Guía de Embarazo, Parto y Lactancia
DGSP 2020	Dirección General de Salud Pública, Consejería de Sanidad<br>Cantabria – 2020	out of scope	Cuidados de Salud durante el embarazo
SERGAS 2019	Servizo Galego de Saúde, Dirección Xeral de Asistencia Sanitaria<br>Galiza – 2019	out of scope	Consulta preconcepcional e de atención ao embarazo normal – Proceso asistencial
NHMRC 2020	Australian Government Department of Health<br>Australia – 2020	out of scope	Clinical Practice Guidelines: Pregnancy Care
RANZCOG 2022	Royal Australian and New Zealand College of Obstetricians and Gynecologists<br>Australia – 2022	out of scope	Best practice statement on routine antenatal assessment in the absence of pregnancy complications
SNS 2014	Ministerio de Sanidad, Servicios Sociales e Igualdad<br>Spain – 2014	out of scope	Guía de práctica clínica de atención en el embarazo y puerperio
SNS 2017	Ministerio de Sanidad, Servicios Sociales e Igualdad<br>Spain – 2017	out of scope	Guía de Práctica Clínica sobre lactancia maternal
KCE 2015	Belgian Health Care Knowledge Centre<br>Belgium – 2015	out of scope	What are the recommended clinical assessments and screening tests during pregnancy?
NHG 2023	Nederlands Huisartsen Genootschap<br>Netherlands – 2023	out of scope	Zwangerschap en kraamperiode
NICE NG201	NICE<br>UK – 2021	out of scope	Antenatal care
NICE CG192	NICE<br>UK – 2020	out of scope	Antenatal and postnatal mental health: clinical management and service guidance

NICE NG194	NICE<br>UK – 2021	out of scope	Postnatal care
WHO 2016	World Health Organization<br>2016	out of scope	WHO recommendations on antenatal care for a positive pregnancy experience
WHO 2015	World Health Organization<br>2015	out of scope	WHO recommendations on health promotion interventions for maternal and newborn health
**REASON FOR EXCLUSION: out of scope – general recommendations on exposure to toxics or environmental factors**			
WHO 2021	World Health Organization<br>2021	out of scope	WHO guideline for the clinical management of exposure to lead
NIOSH 2016	US National Institute for Occupational Safety and Health (NIOSH)<br>US – 2016	out of scope	NIOSH criteria for a recommended standard: occupational exposure to heat and hot environments
USPSTF 2019	US Preventive Services Task Force (USPSTF)<br>US – 2019	out of scope	Screening for Elevated Blood Lead Levels in Children and Pregnant Women: US Preventive Services Task Force Recommendation Statement
NIOSH 1999	US National Institute for Occupational Safety and Health (NIOSH)<br>US – 1999	out of scope	The Effects of Workplace Hazards on Female Reproductive Health
OSHA 2000	Occupational Safety and Health Administration (OSHA)<br>US – 2000	out of scope	Directorate of Technical Support and Emergency Management. Anesthetic Gases: Guidelines for Workplace Exposures
**REASON FOR EXCLUSION: out of scope – general recommendations on occupational health**			
WHO 2022	World Health Organization<br>2022	out of scope	Caring for those who care: guide for the development and implementation of occupational health and safety programmes for health workers
NICE NG212	NICE<br>UK – 2022	out of scope	Mental wellbeing at work
ILO 2000a	International Labour Organization<br>2000	out of scope	C183 - Maternity Protection Convention, 2000 (No. 183)
ILO 2000b	International Labour Organization<br>2000	out of scope	R191 - Maternity Protection Recommendation, 2000 (No. 191)
ILO 1999	International Labour Organization<br>1999	out of scope	Maternity protection at work. Report 87
ILO 1995	International Labour Organization<br>1995	out of scope	Maternity and work (Conditions of work digest, vol.13/1994)
ANSES 2016	Agence Nationale Sécurité Sanitaire Alimentaire Nationale (ANSES)<br>France – 2016	out of scope	Report on the Assessment of the health risks for professionals exposed to atypical working hours, especially night work
CDC 1998	Centers for Disease Control and Prevention (CDC)<br>US-1998	out of scope	Guideline for Infection Control in Healthcare Personnel, 1998

CDC 2019	Centers for Disease Control and Prevention (CDC)<br>US-2019	out of scope	Infection Control in Healthcare Personnel: Infrastructure and Routine Practices for Occupational Infection Prevention and Control Services (Update of CDC 1998)
CDC 2025	Centers for Disease Control and Prevention (CDC)<br>US-2025	out of scope	Infection Control in Healthcare Personnel: Epidemiology and Control of Selected Infections Transmitted Among Healthcare Personnel and Patients (Update of CDC 2019)
**REASON FOR EXCLUSION: out of scope – lactation**			
ANVISA 2015	Ministério da Saúde<br>Brasil – 2015	out of scope	Guia para implantação de salas de apoio à amamentação para a mulher trabalhadora
WHO 2018	World Health Organization<br>2018	out of scope	Guideline: counselling of women to improve breastfeeding practices
RNAO 2018	Registered Nurses' Association of Ontario<br>Canada – 2018	out of scope	Breastfeeding - promoting and supporting the initiation, exclusivity, and continuation of breastfeeding for newborns, infants, and young children
**REASON FOR EXCLUSION: other reasons**			
RCP 2013 summary	Royal College of Physicians (RCP)<br>UK – 2013	overlap	Pregnancy: occupational aspects of management: concise guidance
ISTAS 2012	Instituto Sindical de Trabajo, Ambiente y Salud (ISTAS)<br>Spain – 2012	overlap	Guía sindical mujeres, trabajos y salud
N/A	American College of Occupational and Environmental Medicine (ACOEM)<br>US – 2011	out of date	Reproductive and developmental hazard management guidance: ACOEM Task Force on Reproductive Toxicology
N/A	American College of Occupational and Environmental Medicine (ACOEM)<br>US – 1996	out of date	ACOEM reproductive hazard management guidelines. American College of Occupational and Environmental Medicine
RCOG 2013	Royal College of Obstetricians and Gynecologists (RCOG)<br>UK – 2013	no recommendations	Chemical Exposures During Pregnancy: Dealing with Potential, but Unproven, Risks to Child Health
